# Design issues with lutetium-177 PSMA-617 registration studies that bias the outcome of the experimental arm reflect an increasing misalignment of contemporary oncology trials with true patient benefit

**DOI:** 10.1038/s44276-024-00065-7

**Published:** 2024-06-24

**Authors:** J. B. Schnog, A. J. Duits, M. J. Samson

**Affiliations:** 1 Department of Hematology-Medical Oncology, Curaçao Medical Center, Willemstad, Curaçao; 2 Curaçao Biomedical & Health Research Institute, Willemstad, Curaçao; 3 Department of Medical Education, Curaçao Medical Center, Willemstad, Curaçao; 4 Institute for Medical Education, University Medical Center Groningen, Groningen, the Netherlands; 5 Red Cross Blood Bank Foundation, Willemstad, Curaçao; 6 Department of Radiation Oncology, Curaçao Medical Center, Willemstad, Curaçao

## Abstract

In the *PSMAfore* randomized controlled trial patients with chemotherapy naïve castrate resistant metastasized prostate cancer (CRPC) progressing after one line of a second-generation androgen receptor signaling inhibitor (ARSI) were randomized to the experimental arm of lutetium-177 PSMA-617 or the control arm of another ARSI. The trial showed an increase in the primary endpoint radiographic progression free survival in the experimental arm. Previously, the VISION trial led to the approval of lutetium-177 PSMA-617 in patients with CRPC progressing after at least 1 second generation ARSI and at least 1 line of chemotherapy with a taxane. We highlight several shortcomings in both trials concerning use of putative surrogate endpoints, control arm treatments not reflective of contemporary standards of care, informative censoring and inappropriate cross-over, that all bias results in favor of the experimental arms. Additional regulatory approval of lutetium-177 PSMA-617 for patients prior to receiving chemotherapy would not only lead to further exposure of patients to a treatment without proper proof of benefit but to unsubstantiated health care spending as well.

## Introduction

Lutetium-177 PSMA-617 is FDA approved for treatment of patients with castrate resistant metastasized prostate cancer (CRPC) progressing after at least one second generation androgen receptor signaling inhibitor (ARSI) and 1 line of taxane based chemotherapy based on a 4-month median overall survival (OS) benefit demonstrated in the VISION randomized controlled trial (RCT) [1] (see Table 1). Recently the results of the industry-sponsored *PSMAfore* study were presented at the European Society of Medical Oncology 2023 meeting, with some of the shortcomings detailed below outlined by the discussant (https://www.urotoday.com/conference-highlights/esmo-2023/esmo-2023-prostate-cancer/147589-esmo-2023-psmafore-discussant-using-clinical-intelligence-to-define-first-line-mcrpc-therapy.html) [2]. In this study, patients with chemotherapy naïve CRPC with positive prostate-specific membrane antigen (PSMA)-avid disease on positron emission tomography and prior treatment with an ARSI, were randomized to the experimental arm of lutetium-177 PSMA-617 or the control arm of a subsequent ARSI (see Table 1). The primary endpoint, increased radiographic progression free survival (rPFS), was met and the drug manufacturer will seek Food and Drug Administration (FDA) approval (https://www.biopharmadive.com/news/novartis-pluvicto-fda-filing-prostate-cancer-psmafore/697626/). Moving Lutetium-177 PSMA-617 to an earlier line targets a larger patient population with a positive trial outcome potentially increasing the market share for the tested drug. However, for potential earlier use the trial should demonstrate that treating patients with CRPC and a positive PSMA scan with lutetium-177 PSMA-617 leads to an improvement in overall survival (OS) and/or quality of life (QOL) as compared to the current best standard of care after progressing on an ARSI, which is chemotherapy. We have several concerns regarding the design of both the *PSMAfore* and VISION trial, the latter recently addressed by Olivier et al., that may bias trial results in favor of the experimental arm [3].

**Table 1:** Characteristics of RCTs with Lu- 177 PSMA-617.

Trial	Phase	Accrual	*n*	Patients	Primary outcome	Control arm	Cross-over	Unbalanced early censoring
VISION	3	2018–2019	831	mCRPC progressed after ARSI and at least 1 line of taxane chemotherapy	rPFS and OS	ARSI, bisphosphonates, radiation therapy, denosumab, glucocorticoids, ADT. Not allowed: chemotherapy, systemic radioisotopes, immunotherapy, investigational agents	No	Lu- 177 PSMA-617: 3% estimated. Control arm: 16.3%.
TheraP	2^a^	2018–2019	200	mCRCP progressed after docetaxel with previous ARSI allowed	Serum PSA reduction ≥50% from baseline	Cabazitaxel, supportive care including ADT.	No	Lu- 177 PSMA-617: 2.0% Control arm: 16.8%
PSMAfore	3	2021–2022	468	mCRPC progressing after 1 line of an ARSI and no previous chemotherapy	rPFS	ARSI, supportive care including ADT. Not allowed: investigational agents, biological products, immunotherapy, cytotoxic chemotherapy, other systemic radioisotopes, PARP inhibitors, hemi-body radiotherapy.	Yes	Lu- 177 PSMA-617: 3.0%. Control arm: 0.9%^b^

*mCRPC* metastatic castration resistant prostate cancer, *rPFS* radiographic progression free survival, *OS* Overall survival, *ARSI* second generation androgen receptor signaling inhibitor, *ADT* androgen deprivation therapy, *PSA* prostate-specific antigen, *PARP* poly-ADP ribose polymerase.

^a^Randomized.

^b^Final publication not available at time of writing (data from: https://www.urotoday.com/conference-highlights/esmo-2023/esmo-2023-prostate-cancer/147587-esmo-2023-psmafore-phase-3-trial-of-177lu-lu-psma-617-in-taxane-naive-patients-with-mcrpc.html (accessed April 2024).

## Concerns regarding *PSMAfore*

First, the *PSMAfore* trial uses the putative surrogate endpoint of rPFS as the primary endpoint. Putative surrogate endpoints are increasingly used in contemporary oncology trials even though they often do not adequately predict OS and/or QOL [4–6]. Furthermore, they are often not adequately reported [7]. In a recent meta-analysis rPFS did not reliably predict OS in CRPC patients treated either with hormonal- or chemotherapy, with no data available for treatment with lutetium-177 PSMA-617 [8]. In the *TheraP* trial, an improvement in rPFS in the Lutetium-177 PSMA-617 arm did not translate to OS when patients were randomized to Lutetium-177 PSMA-617 or cabazitaxel after most had progression on an ARSI and all were previously treated with docetaxel [9] (see Table 1). Putative surrogate endpoints that do not reliably predict OS are unlikely to predict QOL [10]. Based on the above, the attained primary endpoint of a rPFS increment in the *PSMAfore* trial is unlikely to reflect clinical significance.

Second, the offered control arm treatment in *PSMAfore* is not reflective of the contemporary standard of care. Control arms not reflective of the standard of care occur regularly in oncological trials, which usually favors the outcome of the experimental arm [11, 12]. An empirical analysis of control arm quality has shown a third of genitourinary oncology randomized trials employing suboptimal control arm treatment [11]. Patients could receive enzalutamide after abiraterone (or another ARSI) or vice-versa, which will not lead to substantial health benefit and is not a recommended treatment, as outcome is generally poor [13–15]. A similar recent example is seen in the Profound RCT [16, 17]. Patients with metastatic CRPC with homologous recombination repair gene mutations were randomized to olaparib (experimental arm) or the criticized suboptimal control arm enzalutamide after abiraterone or vice versa [18]. In the National Comprehensive Cancer Network (NCCN) guideline the preferred treatment after an ARSI is docetaxel (https://www.nccn.org/professionals/physician_gls/pdf/prostate.pdf). In the European Society of Medical Oncology (ESMO) guidelines the use of abiraterone after enzalutamide or vice versa is not recommended [19]. ARSI rechallenge has recently been addressed as a recurring concern in the control arms of several prostate cancer registration trials in which, for establishing new treatments, the experimental arm should be compared to the best standard of care [20]. In *PSMAfore*, patients are stratified as being asymptomatic, mildly symptomatic or symptomatic (https://classic.clinicaltrials.gov/ct2/show/NCT04689828). One could question how scientifically sound and ethical it is to offer a treatment without expected health benefit, and is discouraged in societal guidelines, to a (mildly) symptomatic patient in the control arm, thereby delaying the proven effective next line of therapy with docetaxel. Furthermore, given the *PSMAfore* inclusion criteria (Eastern Cooperative Oncology Group performance status 0-1, adequate bone marrow reserve, renal and hepatic function) it seems that most included patients should be eligible for docetaxel (it is very unlikely that with only 3.0% of control arm patients harboring liver metastases many would have had an elevated total bilirubin concentration precluding docetaxel treatment) (https://classic.clinicaltrials.gov/ct2/show/NCT04689828, https://www.accessdata.fda.gov/drugsatfda_docs/label/2012/201525s002lbl.pdf, http://www.bccancer.bc.ca/drug-database-site/Drug%20Index/Docetaxel_monograph.pdf). The *PSMAfore* trial design therefore biases in favor of the experimental arm outcome by the allowed treatment in the control arm.

Third, the trial has ensured cross-over to Lutetium-177 PSMA-617 after progressing on the control arm. Inappropriate cross-over (cross-over to the experimental drug of the control arm for which the fundamental efficacy has not been proven or withholding cross-over to the experimental drug when it’s efficacy has been proven in the following treatment line) is recognized as a frequent problem in oncology RCT’s that confounds interpretation and raises ethical concerns [11, 21, 22]. Crossing over to Lutetium-177 PSMA-617 after progressing on the control arm assumes that the fundamental efficacy of Lutetium-177 PSMA-617 after ARSI retreatment is proven to be superior to treatment with chemotherapy. In the VISION trial, Lutetium-177 PSMA-617 has shown a statistically significant OS *after* 1 line of chemotherapy [1], but not as compared to second line chemotherapy. As mentioned, the *TheraP* trial failed to show an improvement in OS of Lutetium-177 PSMA-617 over cabazitaxel after progression on ARSI and docetaxel in CRPC [9]. Conceding the fact that this was a phase 2 trial, underpowered for detecting an effect on OS, this lack of OS benefit was observed even with informative censoring occurring early in the cabazitaxel arm (17 patients censored of 101 at risk), which likely favored the Lutetium-177 PSMA-617 arm outcome (2 patients censored of 99 at risk). This, together with several shortcomings in the VISION trial detailed below, suggests that even proof of the fundamental efficacy of Lutetium-177 PSMA-617 after chemotherapy is debatable. Therefore, it is likely that cross-over to Lutetium-177 PSMA-617 after progressing on the control arm in the *PSMAfore* trial will also bias the results to the experimental arm. In the control arm of the *PSMAfore* trial, patients are being deprived of the current standard of care (docetaxel) and upon progression are crossed over to Lutetium-177 PSMA-617, a treatment without proper proof of efficacy. Therefore, any possible OS gain with longer follow-up of patients in the *PSMAfore* trial could be attributable, for a large part (if not only), to twice withholding the recommended life prolonging therapy (docetaxel) in the control arm. Cross-over in the *PSMAfore* trial therefore biases in favor of the experimental arm outcome. To appreciate this point, issues with the VISION trial that have recently been debated need to be addressed [3, 23, 24].

## Concerns regarding VISION

In the VISION RCT, Lutetium-177 PSMA-617 was studied in metastasized CRPC patients who progressed after at least one ARSI and 1 line of taxane based chemotherapy. The study demonstrated a 4-month median overall survival (OS) benefit in Lutetium-177 PSMA-617 treated patients as compared to standard of care [1]. Two important points should be noted.

First, in the control arm of VISION, patients that previously received only first line chemotherapy (docetaxel) could participate if the physician deems them unsuitable to receive 2nd line chemotherapy with cabazitaxel (e.g. frail or intolerant), which was a proven life-prolonging treatment option after failing docetaxel (https://clinicaltrials.gov/study/NCT03511664) [25]. Treatment with enzalutamide or abiraterone was allowed, as were radiotherapy, denosumab and glucocorticoids. More than half of patients on the control arm had already received both abiraterone and enzalutamide and would thus be randomized to a treatment with an ARSI on which they had already progressed [3]. During the accrual phase of VISION, cabazitaxel was shown to improve OS over abiraterone after enzalutamide or vice versa in a RCT [25]. In VISION, 61.8% of patients in the control arm (*n* = 173) were cabazitaxel naïve, and post protocol 53 of 97 patients that received medical treatment in the control arm ultimately received cabazitaxel (10 of 97 docetaxel) with 82 of 155 patients in the experimental arm receiving cabazitaxel post-protocol (27 of 155 docetaxel). This very likely would have been a higher proportion and absolute number had the trial appropriately allowed treatment with cabazitaxel in the control arm, and casts doubt as to whether included patients were truly unsuitable to receive chemotherapy. As stated, currently in the NCCN guideline the preferred treatment after prior docetaxel *and* prior ARSI is cabazitaxel or a docetaxel re-challenge, and ESMO guidelines discourage the use of abiraterone after enzalutamide or vice versa (https://www.nccn.org/professionals/physician_gls/pdf/prostate.pdf) [19].

Second, with VISION being an open label study, informative censoring occurred in 56% of the patients in the control arm at the start of the study, ultimately reduced to 16.3% with approximately 3% estimated to be censored in the Lutetium-177 PSMA-617 arm at the first time point [1]. One of the reasons for early censoring to occur in the control arms of open label studies has been hypothesized to be due to patient disappointment [26]. Characteristics of such patients likely differ from patients that remain on study. It has been postulated that they may be healthier and/or are more likely able to acquire better treatment options outside of the trial’s control arm [3, 26]. As a consequence, it is possible that the control arm of VISION is characterized by a more vulnerable population that could bias the outcome of the experimental arm of the study over the control arm [3, 27]. Such a rate of informative censoring was a reason for the FDA to disapprove drugs in the past as a RCT is in such a case *de facto* no longer considered randomized [3]. Indeed, in a modeling experiment of Kaplan–Meier estimates of an RCT characterized by approximately 7% informative censoring, by assuming best- and worst-case scenarios in the censored patients, results of the trial outcome could shift from positive to negative [28, 29]. Drugs without anti-tumor activity can appear to show an artifactual PFS benefit solely by informative censoring, and the importance of recognizing the increasing occurrence thereof as a potential cause of inflated surrogate endpoint improvements in oncology RCT’s has recently been addressed [30–32].

## Untoward consequences of potential Lutetium-177 PSMA-617 approval as per the *PSMAfore* trial

Merely being an active drug (generating a tumor response) does not ensure efficacy (increasing OS and/or QOL in a RCT). As an active drug, Lutetium-177 PSMA-617 may offer clinical benefit for patients in certain scenarios, for example, in those who decline chemotherapy or have contraindications. However, based on the RCTs discussed above, efficacy in patients after treatment with an ARSI and one line of chemotherapy, or prior to chemotherapy, has not been satisfactorily proven. The *PSMAfore* trial, as the VISION trial, are reflective of a system allowing patient accrual on clinical trials with design limitations. The issues addressed such as putative surrogate endpoints, control arms not reflective of standard of care, inappropriate cross-over and informative censoring are increasingly recognized limitations of contemporary oncology trials that all bias outcome in favor of the experimental arm. Failure to recognize such trial design shortcomings by drug regulatory agencies allows persistence of such shortcomings at the level of trial design, medical ethical review boards approval, and professional society guidelines inclusion. This all contributes to the advent of often costly new cancer treatments or indications with marginal (if any) benefit for patients and a substantial impact on the available healthcare budgets [33, 34].

VISION has led to regulatory agency approval with subsequent guideline incorporation [19]. Based on the shortcomings of VISION as detailed above, the inclusion of treatment with Lutetium-177 PSMA-617 of CRPC patients after an ARSI and one line of chemotherapy is, in our opinion, debatable. In our local oncology practice in a small Island developing state (SIDS), it is not offered as a treatment option (for which patients would also need to be referred abroad adding costs to an already expensive treatment). Based solely on the improvement in rPFS, potential FDA approval of Lutetium-177 PSMA-617 in chemotherapy naïve patients based upon the *PSMAfore* trial would further complicate our day-to-day care for patients with prostate cancer. Patients understandably seek to attain the newest, often media-hyped, treatments. Especially in SIDS and low-middle income countries where such treatment options may not be readily available and may often not be reimbursed, patients may risk catastrophic personal expenditure. With costs of cancer care reaching unsustainable levels in even high-income countries, such treatments may not prove attenable. Physicians aware of the lack of proven benefit of Lutetium-177 PSMA-617 risk losing the trust of their patients when trying to protect them from an unproven treatment and society of unsubstantiated spending [35]. Clinical trial design and regulatory agency approvals should align primarily with patient benefit and not commercial or academic gain [33, 34, 36].
